# Ultrasound-Guided Diagnosis of Intermetatarsal Bursitis Mimicking Morton’s Neuroma: A Case Report

**DOI:** 10.7759/cureus.86184

**Published:** 2025-06-17

**Authors:** Abderrahim Lachhab, Yassine Benghali, Mohamed Maroc, Yassin Nkhili, Ahmed Amine El Oumri

**Affiliations:** 1 Faculty of Medicine, Mohammed First University, Oujda, MAR; 2 Physical Medicine and Rehabilitation, Centre Hospitalier Universitaire Mohammed VI Oujda, Oujda, MAR; 3 Physical Medicine and Rehabilitation, Mohammed VI University Hospital, Oujda, MAR

**Keywords:** forefoot pain, intermetatarsal bursitis, metatarsalgia, morton’s neuroma, ultrasonography

## Abstract

Metatarsalgia, a common cause of foot pain, significantly impairs quality of life and increases the risk of falls. Differentiating its underlying causes, such as Morton’s neuroma and intermetatarsal bursitis, is crucial due to their distinct pathophysiology and management strategies. Clinical diagnosis can be challenging due to overlapping symptoms, underscoring the value of ultrasonography. This case report describes a 60-year-old woman with persistent metatarsalgia in the second and third interdigital spaces. While clinical findings suggested Morton’s neuroma (positive Mulder test), ultrasound revealed a hypoechoic fluid collection measuring 8 × 4 mm with a thickened bursal wall, indicative of intermetatarsal bursitis, and, notably, no nerve thickening. This led to the exclusion of Morton’s neuroma. The patient reported a pre-treatment Visual Analog Scale pain score of 8/10, which significantly improved to 2/10 following a steroid infiltration and custom insoles. This case highlights the importance of ultrasonography in accurately diagnosing metatarsalgia by differentiating between conditions with similar clinical presentations, thereby guiding appropriate treatment and improving patient outcomes.

## Introduction

Metatarsalgia, a common cause of forefoot pain, is a widespread issue that significantly hinders an individual’s independence and quality of life, predisposing them to locomotor disability, impaired balance, and an increased risk of falls [1,2]. Accurately differentiating between its various underlying causes is crucial, especially when symptoms and physical examination findings are similar. This is because the underlying mechanisms and pathophysiology differ, significantly impacting the condition’s course and the need for appropriate management [3-5]. The limitations of clinical diagnosis based solely on symptoms and physical examination findings highlight the critical role of ultrasonography as a valuable tool in identifying the underlying cause of metatarsalgia [6].

Distinguishing intermetatarsal bursitis from Morton’s neuroma presents a significant diagnostic challenge due to their overlapping clinical features. Both conditions commonly cause pain in the forefoot, often localized to the interdigital spaces, and can present with paresthesia or a feeling of “walking on a pebble.” The Mulder test, a common clinical maneuver, can be positive in both, further contributing to diagnostic confusion [7,8]. This diagnostic ambiguity is clinically critical because their underlying pathophysiology differs, leading to distinct treatment approaches. For instance, intermetatarsal bursitis is typically managed conservatively with steroid injections, orthotics, and anti-inflammatory measures, while symptomatic Morton’s neuroma may ultimately require surgical excision [9]. Misdiagnosis can lead to ineffective treatments, prolonged pain, and unnecessary interventions.

Several prior studies and case reports have highlighted this diagnostic confusion and the distinct ultrasound characteristics of intermetatarsal bursitis and Morton’s neuroma. For example, some studies detail cases of misdiagnosis where patients initially treated for one condition were later found to have the other, underscoring the clinical consequences of incorrect diagnosis [7,8]. Ultrasonography can differentiate between these conditions by revealing a hypoechoic, often compressible fluid collection with a thickened bursal wall in intermetatarsal bursitis, contrasting with the well-defined, ovoid, hypoechoic mass characteristic of Morton’s neuroma, which often shows nerve thickening [10]. This case report underscores the significance of ultrasonography in accurately diagnosing metatarsalgia by ruling out differential diagnoses, particularly emphasizing that intermetatarsal bursitis can mimic Morton’s neuroma, preventing potential misdiagnosis and guiding appropriate management.

## Case presentation

**Figure 1:**
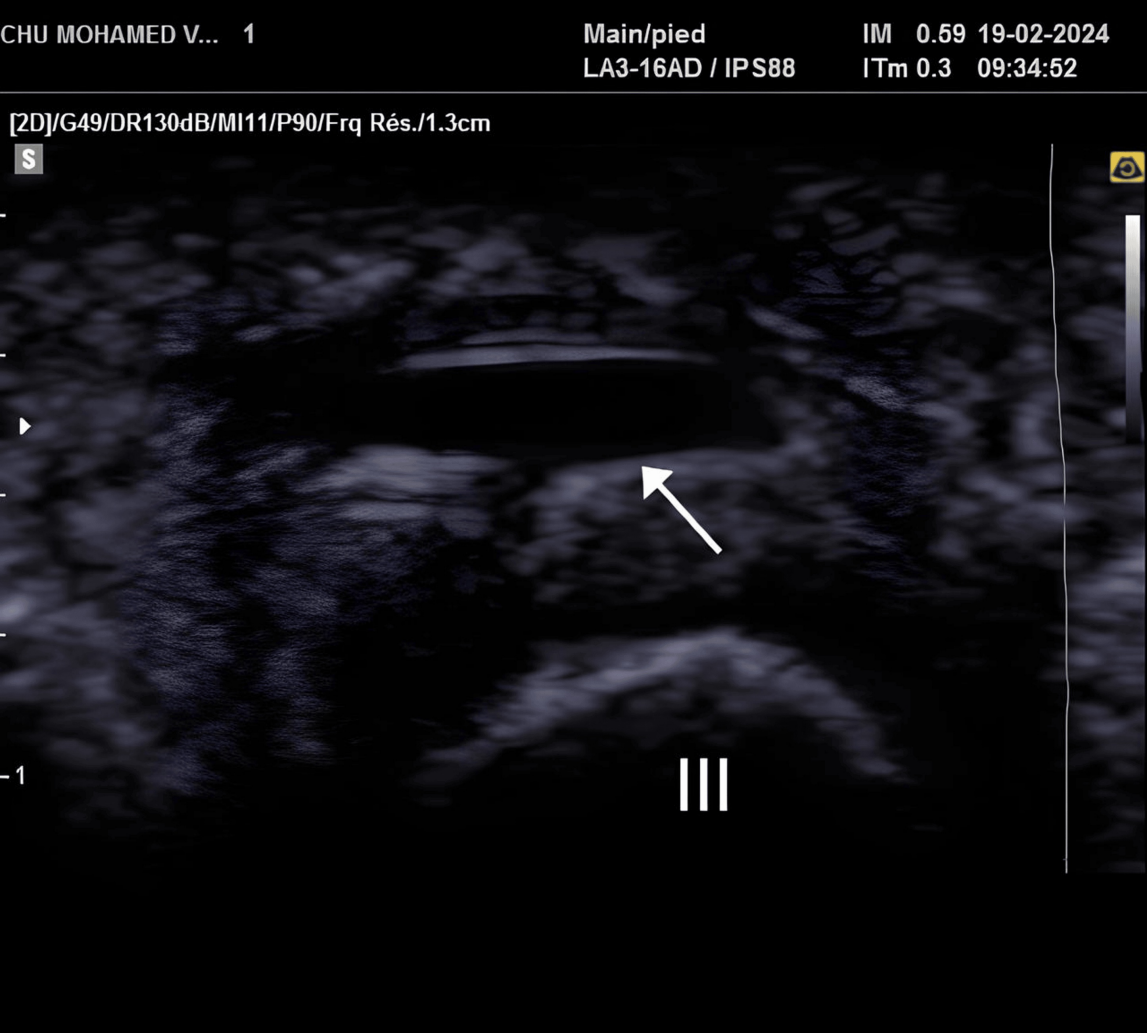
Axial view showing intermetatarsal bursa in the space between the second and third metatarsals. White arrow: intermetatarsal bursa. III: third metatarsal.

**Figure 2:**
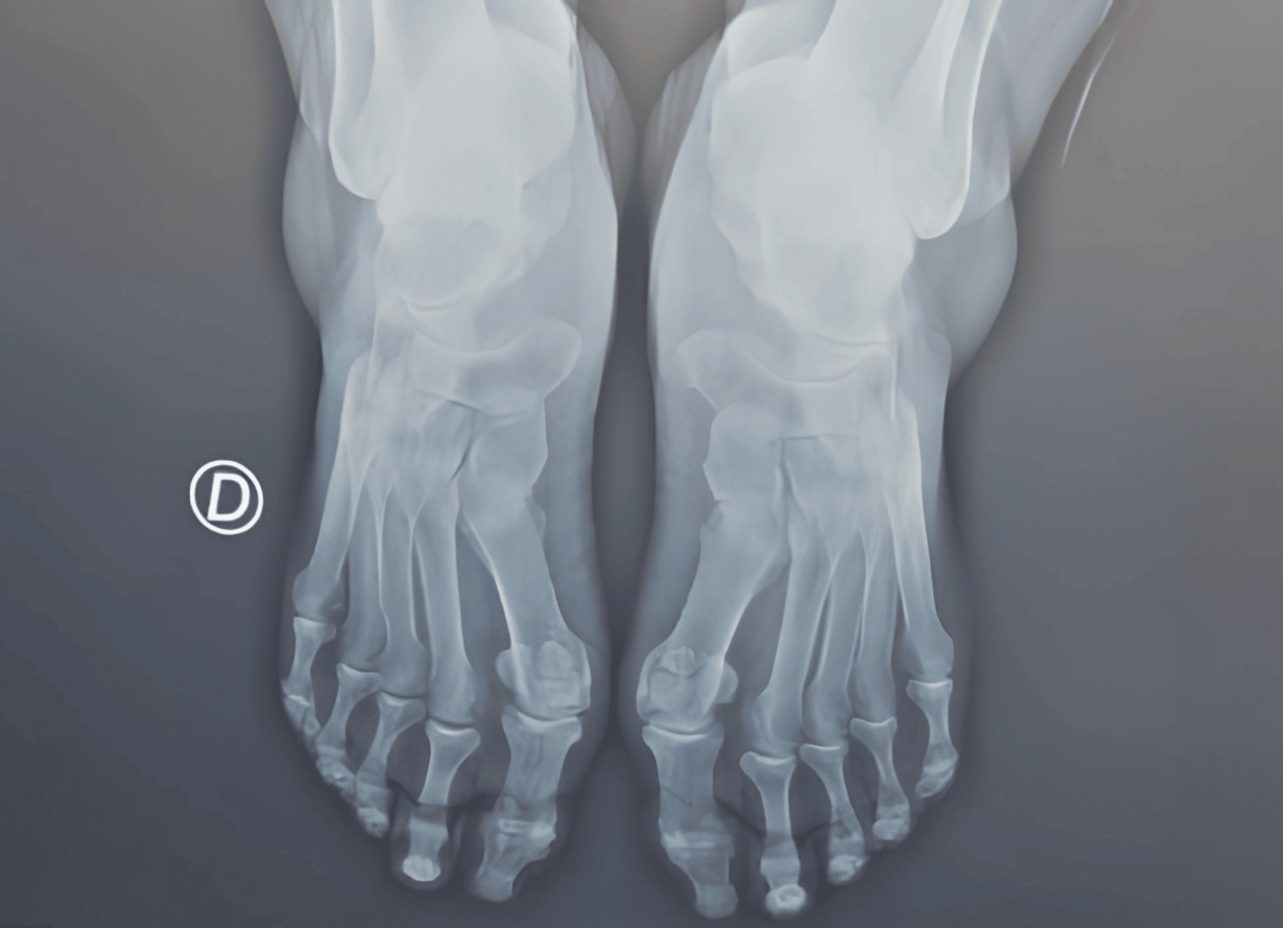
Bilateral hallux valgus X-ray. D: right foot.

A 60-year-old woman with a history of ovarian cancer surgery presented with persistent metatarsalgia for approximately one year. The pain was localized to the second and third interdigital spaces, described as an electric shock, and was particularly aggravated during walking, running, or wearing tight shoes. It was also accompanied by a foreign body sensation. Inspection revealed plantar subluxation of the metatarsal heads and intense pain, rated 8/10 on the Visual Analog Scale (VAS), which was exacerbated by pressure. A positive Mulder’s test initially suggested Morton’s neuroma. However, further investigations provided crucial differentiation. An ultrasound revealed a hypoechoic, oval-shaped fluid collection measuring approximately 8 × 4 mm, with a thickened bursal wall and no evidence of power Doppler signal, confirming the absence of active synovitis. Crucially, the ultrasound also showed no evidence of nerve thickening or neuroma formation (Figure 1). Concomitantly, normal blood tests were noted, including C-reactive protein, erythrocyte sedimentation rate, and rheumatoid factor. An X-ray revealed a slight hallux valgus but no significant intermetatarsal space narrowing or abnormal metatarsal alignment (Figure 2). Based on this comprehensive clinical and complementary examination, Morton’s neuroma was effectively ruled out in favor of intermetatarsal bursitis. The patient received an infiltration of 1 mL of Diprosten steroids. Additionally, she was prescribed insoles with retro-capital support. Follow-up at one month post-treatment demonstrated significant improvement, with her pain score reduced to 2/10 on the VAS.

## Discussion

Metatarsalgia often presents a diagnostic challenge due to the overlapping symptoms of various underlying conditions, with Morton’s neuroma and intermetatarsal bursitis being two frequent sources of forefoot pain [7]. Both conditions can cause sharp, stinging forefoot pain that worsens with walking, as well as numbness or a foreign body sensation in the toes adjacent to the affected area [8-10]. This case report highlights the diagnostic dilemma these conditions pose and underscores the critical role of advanced imaging in achieving an accurate diagnosis.

Initially, the patient’s symptoms, characterized by forefoot pain and paresthesia, strongly suggested a Morton’s neuroma, a common thickening of the nerve structure between the metatarsals, typically the second and third [11]. The pathogenesis of Morton’s neuroma is generally attributed to chronic pressure or repetitive microtrauma, leading to fibrosis around a digital nerve [11]. However, in this case, the clinical presentation alone, while suggestive of neuroma, was insufficient for a definitive diagnosis.

The diagnostic shift from suspected Morton’s neuroma to intermetatarsal bursitis in our patient exemplifies a crucial diagnostic challenge. Intermetatarsal bursitis, an inflammation of the bursae situated between the metatarsal bones, is often overlooked or misdiagnosed due to its symptomatic mimicry of Morton’s neuroma [12]. These bursae are located dorsally to the deep transverse intermetatarsal ligament, adjacent to the common plantar digital nerve, particularly in the second and third web spaces [13]. While blunt trauma is a common cause, it is also important to consider inflammatory arthritis, such as rheumatoid arthritis or gout, as an underlying etiology [12,13]. Our patient’s presentation of forefoot pain, initially pointing toward a nerve-related issue, could also be explained by the inflammatory nature of bursitis. Furthermore, the relationship between intermetatarsal bursitis and Morton’s neuroma is a subject of ongoing debate. Some theories suggest that a swollen and inflamed intermetatarsal bursa can compress the adjacent neurovascular bundle, potentially leading to the development of a neuroma [14-16]. Another hypothesis proposes that inflammation within the bursa directly triggers fibrotic changes in the nearby nerve, ultimately contributing to neuroma formation [13]. This case, therefore, serves as a valuable reminder for clinicians to consider intermetatarsal bursitis in the differential diagnosis of metatarsalgia, even when classic neuroma symptoms are present. Identifying subtle clinical clues to differentiate between the two without imaging can be difficult, emphasizing the need for advanced diagnostic tools.

Ultrasound proved to be the pivotal diagnostic tool in this case, enabling the accurate differentiation between intermetatarsal bursitis and Morton’s neuroma. While MRI can differentiate between the two, with neuromas showing an intermediate T2 signal and bursitis a hyperintense T2 signal [17], ultrasound offers distinct advantages due to its widespread availability, cost-effectiveness, and the ability for dynamic examination [18]. In our patient, ultrasound imaging revealed a well-defined, anechoic (fluid-filled) lesion between the metatarsal heads, characteristic of intermetatarsal bursitis, rather than the hypoechoic, nodular thickening expected with a Morton’s neuroma [19]. Crucially, the dynamic ultrasound examination with localized compression from the opposite side of the intermetatarsal space demonstrated the characteristic deformability and collapse of the lesion, further confirming it as a bursa [19]. In contrast, a Morton’s neuroma would typically retain its well-defined, nodular appearance under such pressure [19]. This dynamic assessment was instrumental in guiding the final diagnosis and, subsequently, the treatment approach. The absence of neural thickening further supported the diagnosis of bursitis over neuroma.

This case highlights several key learning points for clinicians evaluating metatarsalgia. First, while clinical history and physical examination are crucial initial steps, they may not always be sufficient for a definitive diagnosis due to the symptomatic overlap between Morton’s neuroma and intermetatarsal bursitis. Second, ultrasound imaging is an invaluable tool for differentiating these conditions, offering not only diagnostic accuracy but also the ability for dynamic assessment, which is critical in identifying the fluid-filled nature and deformability of bursal lesions. This can significantly improve diagnostic accuracy and help avoid unnecessary or ineffective treatments. Finally, this case serves as a reminder that intermetatarsal bursitis can closely mimic Morton’s neuroma, and a thorough diagnostic workup, especially with imaging, is essential for appropriate patient management and to ensure the most effective treatment plan is implemented.

## Conclusions

This case report highlights the indispensable role of ultrasonography in accurately diagnosing the etiology of metatarsalgia. While clinical presentations of conditions such as Morton’s neuroma and intermetatarsal bursitis frequently overlap, leading to diagnostic challenges, ultrasound imaging offers a definitive means to differentiate these pathologies. By clearly visualizing the underlying anatomical abnormalities, such as a fluid collection and thickened bursal wall versus nerve thickening, ultrasonography allows for a precise diagnosis. This precision is paramount as it enables clinicians to tailor treatment strategies specifically to the underlying cause, preventing misdiagnosis and ineffective interventions, ultimately leading to significantly improved patient outcomes. We advocate for the broader integration of ultrasonography in the diagnostic pathway for metatarsalgia to resolve similar diagnostic dilemmas and ensure optimal patient management.
